# Inhibition of Phosphatidylcholine-Specific Phospholipase C Interferes with Proliferation and Survival of Tumor Initiating Cells in Squamous Cell Carcinoma

**DOI:** 10.1371/journal.pone.0136120

**Published:** 2015-09-24

**Authors:** Serena Cecchetti, Ileana Bortolomai, Renata Ferri, Laura Mercurio, Silvana Canevari, Franca Podo, Silvia Miotti, Egidio Iorio

**Affiliations:** 1 Department of Cell Biology and Neurosciences, Istituto Superiore di Sanità, Rome, Italy; 2 Department of Experimental Oncology and Molecular Medicine, Fondazione IRCCS Istituto Nazionale dei Tumori, Milan, Italy; Second University of Naples, ITALY

## Abstract

**Purpose:**

The role of phosphatidylcholine-specific phospholipase C (PC-PLC), the enzyme involved in cell differentiation and proliferation, has not yet been explored in tumor initiating cells (TICs). We investigated PC-PLC expression and effects of PC-PLC inhibition in two adherent (AD) squamous carcinoma cell lines (A431 and CaSki), with different proliferative and stemness potential, and in TIC-enriched floating spheres (SPH) originated from them.

**Results:**

Compared with immortalized non-tumoral keratinocytes (HaCaT) A431-AD cells showed 2.5-fold higher PC-PLC activity, nuclear localization of a 66-kDa PC-PLC isoform, but a similar distribution of the enzyme on plasma membrane and in cytoplasmic compartments. Compared with A431-AD, A431-SPH cells showed about 2.8-fold lower PC-PLC protein and activity levels, but similar nuclear content. Exposure of adherent cells to the PC-PLC inhibitor D609 (48h) induced a 50% reduction of cell proliferation at doses comprised between 33 and 50 μg/ml, without inducing any relevant cytotoxic effect (cell viability 95±5%). In A431-SPH and CaSki-SPH D609 induced both cytostatic and cytotoxic effects at about 20 to 30-fold lower doses (IC50 ranging between 1.2 and 1.6 μg/ml). Furthermore, D609 treatment of A431-AD and CaSki-AD cells affected the sphere-forming efficiency, which dropped in both cells, and induced down-modulation of stem-related markers mRNA levels (Oct4, Nestin, Nanog and ALDH1 in A431; Nestin and ALDH1 in CaSki cells).

**Conclusions:**

These data suggest that the inhibition of PC-PLC activity may represent a new therapeutic approach to selectively target the most aggressive and tumor promoting sub-population of floating spheres originated from squamous cancer cells possessing different proliferative and stemness potential.

## Introduction

Squamous cell carcinoma (SCC) represents more than 80% of lower tract gynecological cancers, including vulvar and cervical cancers, which are the second most common neoplasia among women up to 65 years of age and is the most frequent cause of death from gynecological malignancies worldwide. Although characterized by a relative slow growth, SCC has a substantial risk of metastasis, especially in immunosuppressed individuals. Surgery and chemo-radiotherapy showed a survival advantage in patients with cervical cancer, nevertheless, even at early stages with expected good prognosis, up to 30% of patients fail to respond to treatment or develop early (< 6 months) recurrent disease with dismal prognosis [1], indicating that some cervical cancer cells have not been eradicated by current treatments. Therefore, improved targeted therapies and new strategies to increase drug and radiation sensitivity are essential for reducing the mortality of this malignancy.

One emerging model for the development of drug- and radio-resistance suggests the existence within tumors of a pool of self-renewing malignant cells that can generate the full repertoire of tumor cells. A subset of tumor initiating cells (TICs) or cancer stem cells has been initially identified in leukemia [2] and then in a variety of solid tumors and in cultured cancer cell lines of different origins [3–12]. The identification of TICs and the definition of factors that sustain their proliferation represent new challenges to develop more efficient anti-cancer therapies [13,14].

Recent studies begin to support a new developing theory about the mechanisms behind the conversion of normal cells into TICs [15,16]. The capability of cancer cells to undergo a metabolic reprogramming might be the key feature to understand the interplay of molecular mechanisms underlying the conversion of normal cells into the TICs [17].

Although an abnormal choline phospholipid metabolism has recently been proposed as a hallmark of tumor cells and possible target for therapy [18,19] little is known about the choline metabolism of stem cells and its changes during the differentiation process [20,21]. Several studies have shown a link between oncogenic signaling pathways and the phosphatidylcholine cycle responsible for the altered profile of choline-containing metabolites during tumor progression. In this context, we showed that phosphatidylcholine-specific phospholipase C (PC-PLC) is strongly up-regulated in epithelial ovarian and breast carcinoma cell lines, compared with their non-tumoral counterparts [22–26]. The competitive PC-PLC inhibitor tricyclodecan-9-yl-potassium xanthate (D609) [27] blocked the proliferation of ovarian cancer cells [24] preventing these cells from entering the S-phase under growth-factor stimulation without inducing cell death [23] and impaired the highly metastatic MDA-MB-231 cell proliferation, by inducing traits of mesenchymal-to-epithelial differentiation [26]. Although these and other studies pointed to an involvement of PC-PLC activity in the proliferation, differentiation and apoptosis of a variety of mammalian cell systems, including non-tumoral stem cells [28–34], no investigations have as yet been addressed to the characterization and role of this enzyme in TICs.

In the present study, using the A431 cell line, we studied PC-PLC protein expression, subcellular localization and activity along with the effects of its inhibition on adherent cells and, taking advantage of non-adherent floating culture [5] that allows for enrichment of TICs, on sphere-forming cells. To support our findings we extended our study to a non-tumoral keratinocyte cell line HaCaT and to another squamous carcinoma cell line, CaSki, which showed a lower stemness potential than the A431 cell line, as assessed by sphere forming efficiency and aldehyde dehydrogenase (ALDH) enzymatic activity [5]. Overall, our results suggest that PC-PLC acts as a master regulator of sphere cell proliferation and survival and its inhibition might represent a new therapeutic approach to selectively affect the most aggressive and tumor promoting sub-population of floating spheres originated from squamous cancer cells possessing different proliferative and stemness potential.

## Materials and Methods

### Antibodies and reagents

Rabbit polyclonal antibodies (Abs) raised against bacterial (*B*. *cereus*) PC-PLC and selectively cross-reacting with mammalian PC-PLC were obtained and characterized as previously reported [35,36,23,28,30–32]. The following antibodies were used: mouse monoclonal anti-β-actin (Sigma-Aldrich, cat. n°A5441, 1:2000), rabbit polyclonal anti-MAPK (ERK1/2, Sigma-Aldrich, cat n°M5670, 1:10000), mouse monoclonal anti-nucleoporin p62 (BD Biosciences, cat. n°610497, 1:500), mouse monoclonal anti-phospho-MAPK (ERK1/2, Thr202/Tyr204, Cell Signaling, cat. n°9106, 1:2000), mouse monoclonal anti-phospho-EGFR (Tyr1068, Cell Signaling, cat. n°2236, 1:2000), rabbit polyclonal anti-phospho AKT (Ser473, Cell Signaling, cat. n°9271, 1:2000), rabbit monoclonal anti-EGFR (Cell Signaling, cat. n°4267, 1:2000), rabbit polyclonal anti-AKT (Cell Signaling, cat. n°9272, 1:2000). The mouse monoclonal anti-EGFR (clone 108) used for immunofluorescence staining was a kind gift of Dr. P.G. Natali (Istituto Tumori Regina Elena, Rome, Italy). The secondary antibodies Alexa Fluor-594 or -488- F(ab’)_2_ fragments of goat anti-rabbit (cat. n° A-11072, 1:200) and goat anti-mouse (cat. n° A-11017, 1:200) were purchased from Molecular Probes (Life Technologies); horseradish peroxidase-conjugated goat anti-mouse IgG (cat. n° 170–6516, 1:3000) and goat anti-rabbit IgG (cat. n° 170–6515, 1:3000) were from BioRad Laboratories Inc. Triton X-100, tricyclo-decan-9-yl-potassium xanthate (D609), poly-L-lysine and all other chemicals and biochemicals were from Sigma-Aldrich, unless otherwise specified.

### Culture of cell lines

The human keratinocyte cell line HaCaT (kindly provided by Dr. E. Tamborini, INT-Milan, Italy) [37] and the human squamous carcinoma cell lines A431 and CaSki (ATCC, ID CRL-1555 and CRL-1550, respectively) were cultured in adherent condition (AD) in RPMI-1640 medium (Lonza Group Ltd) containing 10% fetal bovine serum (FCS) (Lonza), and 1% glutamine (Lonza) and then incubated at 37°C in atmosphere containing 5% CO_2_. Cell lines were genotyped at the fragment analysis facility of the Istituto Nazionale Tumori, Milano, using Stem Elite ID System (Promega), according to manufacturer’s instructions and ATCC guidelines, and their identity was confirmed. Cells were routinely confirmed to be mycoplasma-free using the Mycoplasma Detection Kit Venor GeM (Minerva Biolabs).

### Preparation and culture of A431 and CaSki spheres (SPH)

Spheres were obtained as described [5]. Briefly, A431- and CaSki-AD cells were plated at limited dilution (1,000/ml) in MEGM BulletKit serum free, supplemented with BPE, 2 ml; hEGF, 0.5 ml; Hydrocortisone, 0.5 ml; GA-1000, 0.5 ml; Insulin, 0.5 ml (Lonza) in Ultra Low Attachment Plates (Corning Inc.) and the subsequent organization of spheres was monitored every 3 days. Spheres were trypsinized with TrypLE Express (Invitrogen, Life Technologies), counted and then re-seeded under the same culture conditions or used for in vitro experiments.

### Confocal Laser Scanning Microscopy (CLSM)

For immunofluorescence analyses, HaCaT and A431 cells were stained with the rabbit-anti-PC-PLC antibody followed by goat anti-rabbit Alexa Fluor secondary antibody (before any fixation process) to selectively detect the protein expression on the plasma membrane, otherwise cells were fixed in 3% paraformaldehyde and permeabilized by Triton X-100 before staining. In some experiments the PC-PLC inhibitor D609 (50 μg/ml) was added 24h after seeding and maintained in the cell culture for further time intervals (24h and 48h) prior to the staining. A431-SPH were seeded on coverslips coated with 10 μg/ml poly-L-lysine (Sigma-Aldrich), fixed, permeabilized and stained as above. The cover glasses were mounted on the microscope slide with Vectashield anti-fade mounting medium containing 4’ 6-diamidino-2-phenylindole (DAPI) (Vector Laboratories). CLSM observations were performed on a Leica TCS SP2 AOBS apparatus (Leica Microsystems), using the confocal software (Leica) and Photoshop CS5 (Adobe Systems).

### Analysis of adherent and sphere cell proliferation

Cells were plated in adherent condition or in suspension as previously described [5]. The proliferation rate was monitored 24h and 48h after D609 treatment (dose range from 1.5 to 50 μg/ml, corresponding to 5.6 to 187 μM) by counting live and dead cells by Trypan blue exclusion assay, both under a microscope and on the automated cell counter Contess (Invitrogen); each experiment contains 3 replicates of each tested dose of D609 and the experiments were repeated at least twice. The relative percentage of live and dead cells was calculated based on the sum of live and dead cells at each time point and dose. The percentage of reduction in the proliferation rate in D609-treated cells in comparison with untreated samples was evaluated at 24h and 48h using this formula: 100%—[(number of treated cells/ number of untreated cells) x 100].

### Western blotting analyses

For PC-PLC protein expression studies, cells were lysed in a RIPA buffer (150 mM NaCl, 50 mM Tris-Cl, pH 7.5, 1% Nonidet P-40, 0.5% sodium deoxycholate, 0.1% SDS) containing the complete protease inhibitor cocktail (Hoffman-La Roche Ltd). Cytoplasmic and nuclear fractions were isolated using a nuclear extract kit (Active Motif), following the manufacturer’s instructions. Each fraction (30 μg protein) and total cell lysates (50 μg protein) were resolved by SDS-PAGE and blotted with different antibodies. To determine the effects exerted by D609 on the phosphorylation status and/or total protein expression of different markers, cells were treated with D609 for 24h and 48h (50 μg/ml or 1.5 μg/ml, as reported) and then lysed in the buffer described above adding phosphatase inhibitors. Protein concentrations were determined by Bradford’s protein assay (Bio-Rad Laboratories). Blots were developed using Immobilion Western (Millipore), images were captured by BioSpectrum Imaging System 810 (UVP) and densitometric analysis of specific protein bands were performed with Image Studio Lite software (LI-COR Biosciences). Results (mean ± SD of three independent experiments) were expressed: i) as relative optical densities of phospho-protein levels (p-EGFR, or p-ERK1/2, or pAKT) normalized to the total protein level (total EGFR, or total ERK, or total AKT); ii) as fold change relative to the optical density of PC-PLC protein level in A431-AD vs A431-SPH cells, or in A431-AD vs CaSki-AD cells normalized to β-actin or nucleoporin (for nuclear fractions).

### In vitro PC-PLC enzymatic activity assay

PC-PLC activity was determined in total lysates of HaCaT or A431-AD cells harvested at early confluence, or on A431-SPH cells at 7 days of culture in suspension, using the Amplex Red phosphatidylcholine-specific phospholipase C assay kit (Molecular Probes) and discriminating the PC-PLD contribution, according to the modifications reported by our group [23,28,32].

### Sphere forming efficiency (SFE) analysis

A431-AD and CaSki-AD cells were treated for 24h and 48h with D609 (50 μg/ml) as described, collected and seeded in MEGM BulletKit serum free (Lonza) at 1 cell/well in 96 wells low-attachment plate (Corning). After one week, the number of spheres was counted and the sphere forming efficiency (SFE) evaluated as previously reported [5], using the formula: [number of spheres/number of seeded cells] x 100.

### Real Time PCR

Total RNA was isolated from A431-AD and CaSki-AD cells treated with D609 (50 μg/ml) or left untreated, using the RNAspin Mini Isolation Kit (GE Healthcare). cDNA was obtained by RT-PCR using a high capacity cDNA archive kit (Applied Biosystem). Quantitative RT-PCR was performed by ABI Prism 7900 HT Sequence detection system (Applied Biosystems) by using TaqMan Gold RT-PCR Reagents (Applied Biosystems) and probes for OCT4, NANOG, NESTIN, ALDH1A1 and GAPDH-VIC (endogenous control) all from Applied Biosystems [5]. For relative quantification, we used the ΔCT method (Applied Biosystems). Analyses were performed using data analysis software (SDS software 2.2.2).

### Statistical analysis

Statistical analyses were performed using GraphPad Prism 3.03 Software (GraphPad Software Inc.). All data were compared by two-tailed unpaired Student t-Test or one-way ANOVA. Differences were considered significant when P<0.05.

## Results

### Comparison of PC-PLC protein expression and activity in the A431 carcinoma cell line and in the immortalized HaCaT keratinocytes

**Fig 1:**
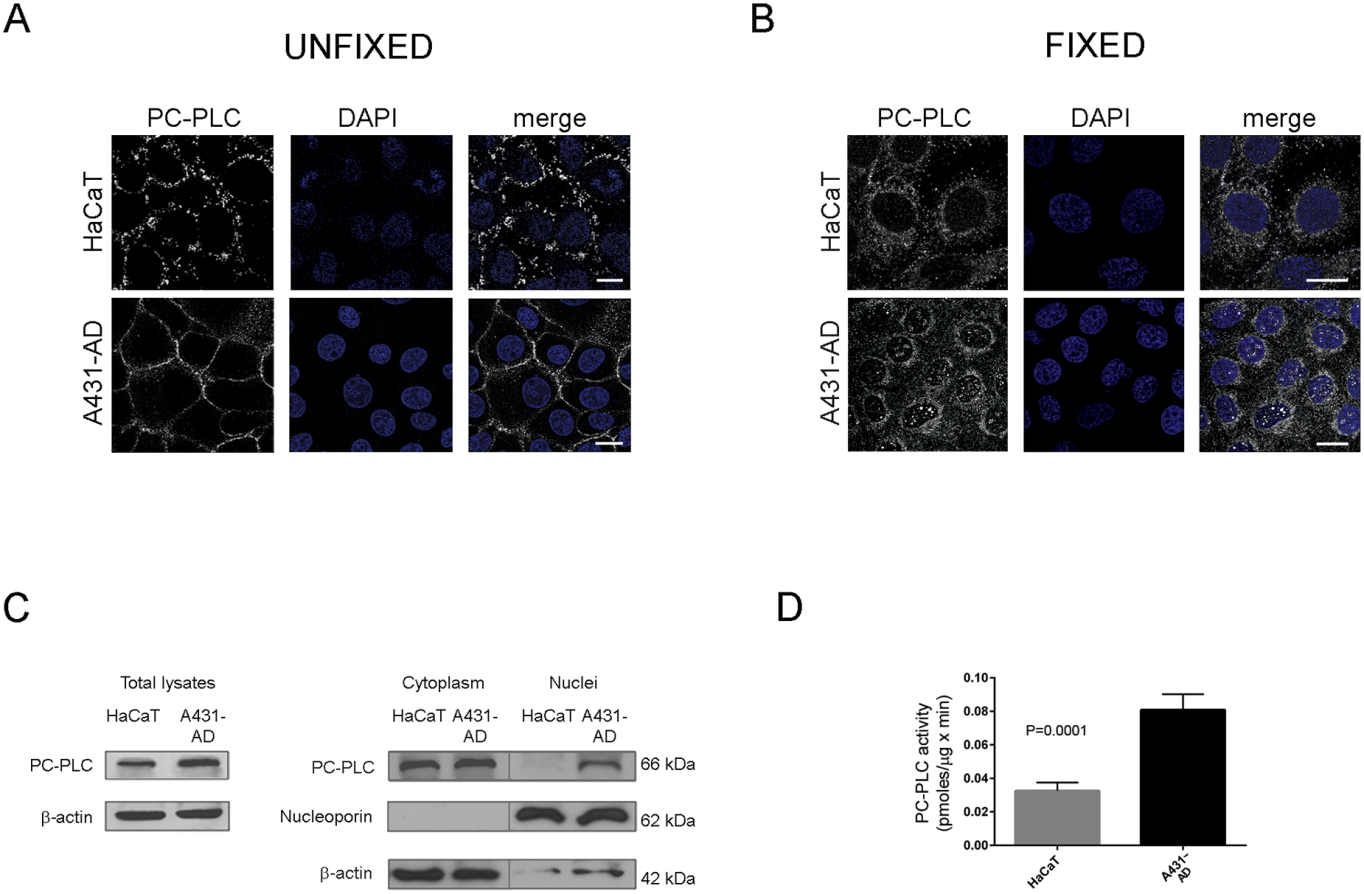
PC-PLC protein expression and activity in the non-tumoral keratinocyte HaCaT and in the A431-AD squamous carcinoma cell lines. Subcellular localization of PC-PLC (grey) on the plasma membrane of unfixed cells (**A**) and in different cellular compartments of fixed and permeabilized cells (**B)** detected by CLSM analyses. Single central optical sections are shown. Nuclei were stained with DAPI (blue). Scale bars, 20 μm. **C**) Western Blot analysis of the relative PC-PLC protein expression in HaCaT and A431-AD total cell lysates (left) and in their cytoplasmic and nuclear fractions (right). Nucleoporin and β-actin were used to ensure the quality of fractions’ separation and protein quantitative loading, respectively. **D)** Absolute PC-PLC activity (pmoles/μg protein x min; mean ± SD, n = 6) measured by Amplex Red assay in total cell lysates. P = 0.0001.

In a study aimed at the characterization of genomic and metabolic profiles of normal keratinocytes and SCC cell lines, we found that the immortalized keratinocyte HaCaT cells exhibit a gene profiling similar to normal keratinocytes (5 samples) and distinct from that of three human SCC (A431, CaSki and SiHa) (Bortolomai I, personal communication), as evaluated by unsupervised hierarchical clustering (see representative dendrogram in S1 Fig). Thus, PC-PLC protein expression was measured in A431-AD cells, as a typical model of SCC, and in HaCaT cells, as a representative model of normal epithelial cells. Confocal Laser Scanning Microscopy (CLSM) analyses of either unfixed or fixed and permeabilized cells showed that PC-PLC is similarly distributed on the plasma membrane (Fig 1A) and in cytoplasmic compartments (Fig 1B) of HaCaT and A431-AD cell lines. PC-PLC also localized as large granules inside the nucleus of A431-AD cancer cells, whereas no PC-PLC-positivity was detected in HaCaT nuclei (Fig 1B). Western blot analysis of sub-cellular fractions further demonstrated the nuclear localization of a PC-PLC isoform (66 kDa) in A431-AD cancer cells only (Fig 1C, right panel). The PC-PLC enzymatic activity, measured with Amplex Red assay in total lysates of cells harvested at early confluence, was about 2.5 fold higher in the A431-AD than in HaCaT cells (P = 0.0001; Fig 1D).

### Effects of the PC-PLC inhibitor D609 on PC-PLC activity, cell proliferation, and protein expression in the HaCaT and A431-AD cell lines

**Fig 2:**
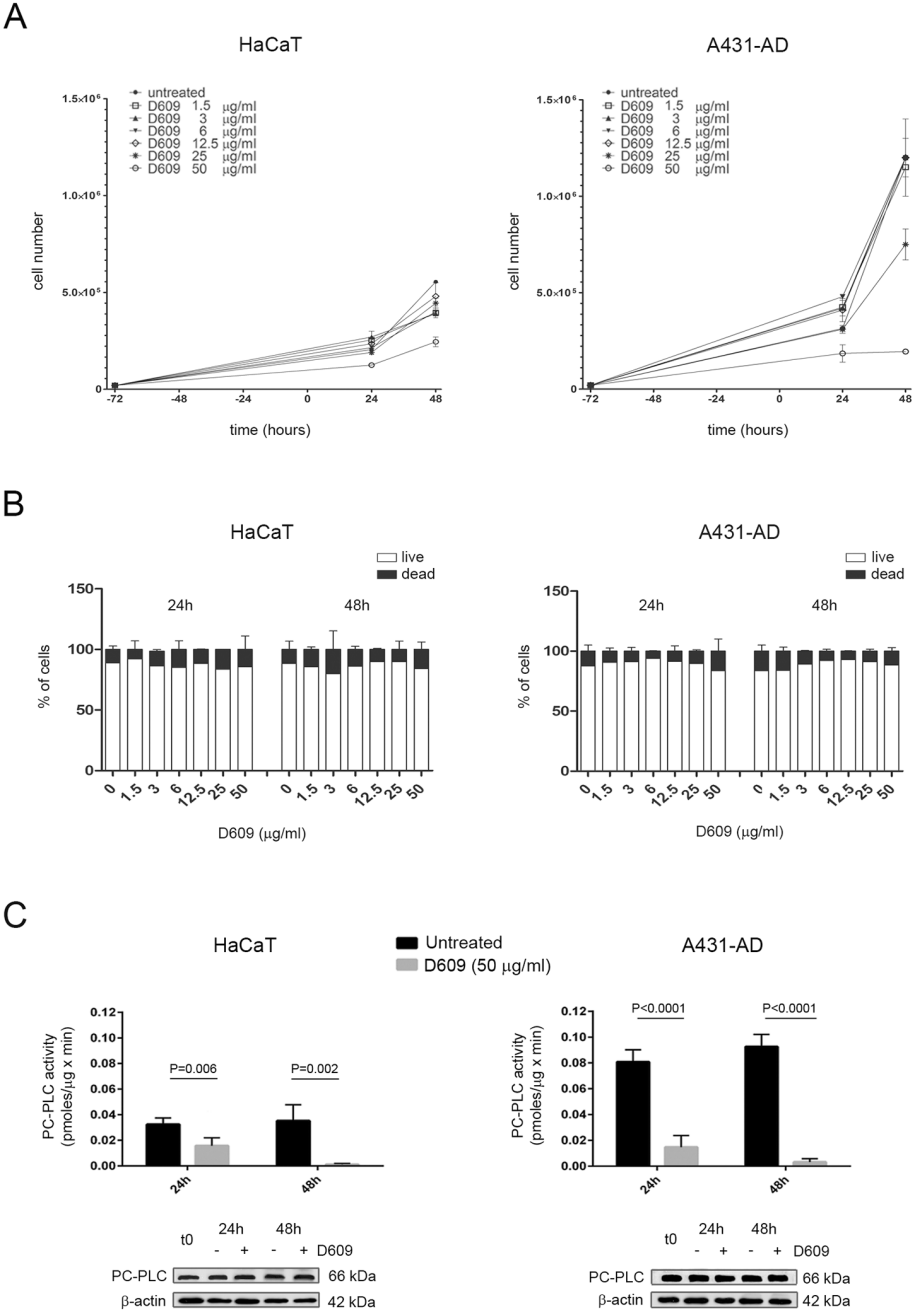
Effects of the PC-PLC inhibitor D609 on PC-PLC activity, PC-PLC protein expression and cell proliferation in HaCaT keratinocytes and in the squamous carcinoma cell line A431-AD. **A**) Proliferation assays performed on cells seeded 72 hours before adding different doses of D609 at t = 0 (● = untreated cells; □ = 1.5 μg/ml, ▲ = 3 μg/ml, ▼ = 6 μg/ml, ◇ = 12.5 μg/ml, * = 25 μg/ml and ○ = 50 μg/ml) and monitored for 24h and 48h afterwards. Cell count (mean ± SD, n = 3) of live cells was obtained by Trypan blue exclusion assays and by automated cell counter, as described in the Materials and Methods section. **B**) Cell counting (mean percentage ± SD, n = 3) of either live (white columns) or dead cells (black columns) measured by Trypan blue exclusion test in the cultures used for the proliferation assays shown in panel A. **C**) Top panel: PC-PLC activity (mean ± SD, n = 3) measured by Amplex Red assay in total lysates of control (untreated = black columns) or 50 μg/ml D609-treated cells (grey columns). Statistical analyses were performed using t-test; HaCaT, P = 0.006 at 24h and P = 0.002 at 48h; A431-AD, P<0.0001 at 24h and at 48h. Bottom panel: Representative Western blot analyses of PC-PLC protein expression performed in total lysates of cells cultured in the presence or absence of 50 μg/ml D609 (n = 3 independent experiments); t0 = untreated cells at 72 hours after seeding; β actin was used as quantitative loading control.

To better understand the functional role of PC-PLC in cancer cells, as well as in their non-tumoral counterpart, we investigated the effects of the PC-PLC competitive inhibitor D609 on cell proliferation by exposing HaCaT and A431-AD cell lines to different doses of the drug. Cells were seeded 72h before adding D609 to the culture medium (t = 0) and the cell proliferation rate was determined 24h and 48h after treatment. In both cell lines D609 at doses up to 12.5 μg/ml induced minimal or no changes in cell proliferation (Fig 2A), while 48h after treatment with 25 μg/ml of D609, a 37.5% reduction of cell proliferation was observed in A431-AD cells only. We found that the 50% of cell proliferation inhibition was achieved at about 50 μg/ml for HaCat and 33.4 μg/ml for A431 cell lines. Continuous exposure to 50 μg/ml of D609 progressively induced cell growth arrest in A431-AD cells, whereas D609-treated HaCaT cells underwent a more limited growth inhibition (83.8% and 56.9% reduction after 48h of treatment, respectively). D609 treatment mainly exerted a cytostatic rather than a cytotoxic effect, as indicated by dead cell counting (restricted to a range of 5–15%, independently from dose and time of treatment) (Fig 2B). This anti-proliferative effect was more evident and remarkable in the A431 cancer cells than in the non-tumoral HaCaT cells. In order to compare the results derived from the further experiments, we decided to use the dose of 50 μg/ml for all the adherent cell lines.

Next, we evaluated PC-PLC activity in HaCaT and A431-AD total cell lysates after treatment with D609 at the dose of 50 μg/ml [23–26,28,30–33]. As reported in Fig 2C (top panel), exposure to D609 for 48h induced in both cell lines an important impairment of PC-PLC activity. Already after 24h of treatment the inhibition of PC-PLC was almost complete and more effective in the A431-AD (87 ± 3%) than in HaCaT cells (50 ± 1%). Western blot analyses (Fig 2A bottom panel) showed that the PC-PLC expression was not modified by D609 treatment at any analyzed time point in either cell line. Additionally, the PC-PLC sub-cellular localization was not changed upon D609 exposure (data not shown).

In our experimental conditions the activity of sphingomyelin synthase (SMS), another enzyme potentially involved in the inhibitory effect of D609 [38], was not affected (S2 Fig). Overall, these results showed that, at the dose of 50 μg/ml, the most relevant inhibitory effect of D609 was targeted against PC-PLC.

### Effect of PC-PLC inhibition on EGFR, ERK and AKT phosphorylation in the HaCat and A431-AD cells

In squamous carcinoma the EGFR gene amplification and the cross-talk between other members of the HER family have been implicated in oncogene-driven processes [39]. Since PC-PLC inhibition had substantial effects on A431-AD squamous cancer cell proliferation, we investigated the changes induced by D609 (50 μg/ml) on the phosphorylation level of EGFR, as well as on the phosphorylation of ERK1/2 and AKT, in order to elucidate whether the inhibitor could interfere with the activation of the two major signaling pathways responsible for cell growth and survival.

**Fig 3:**
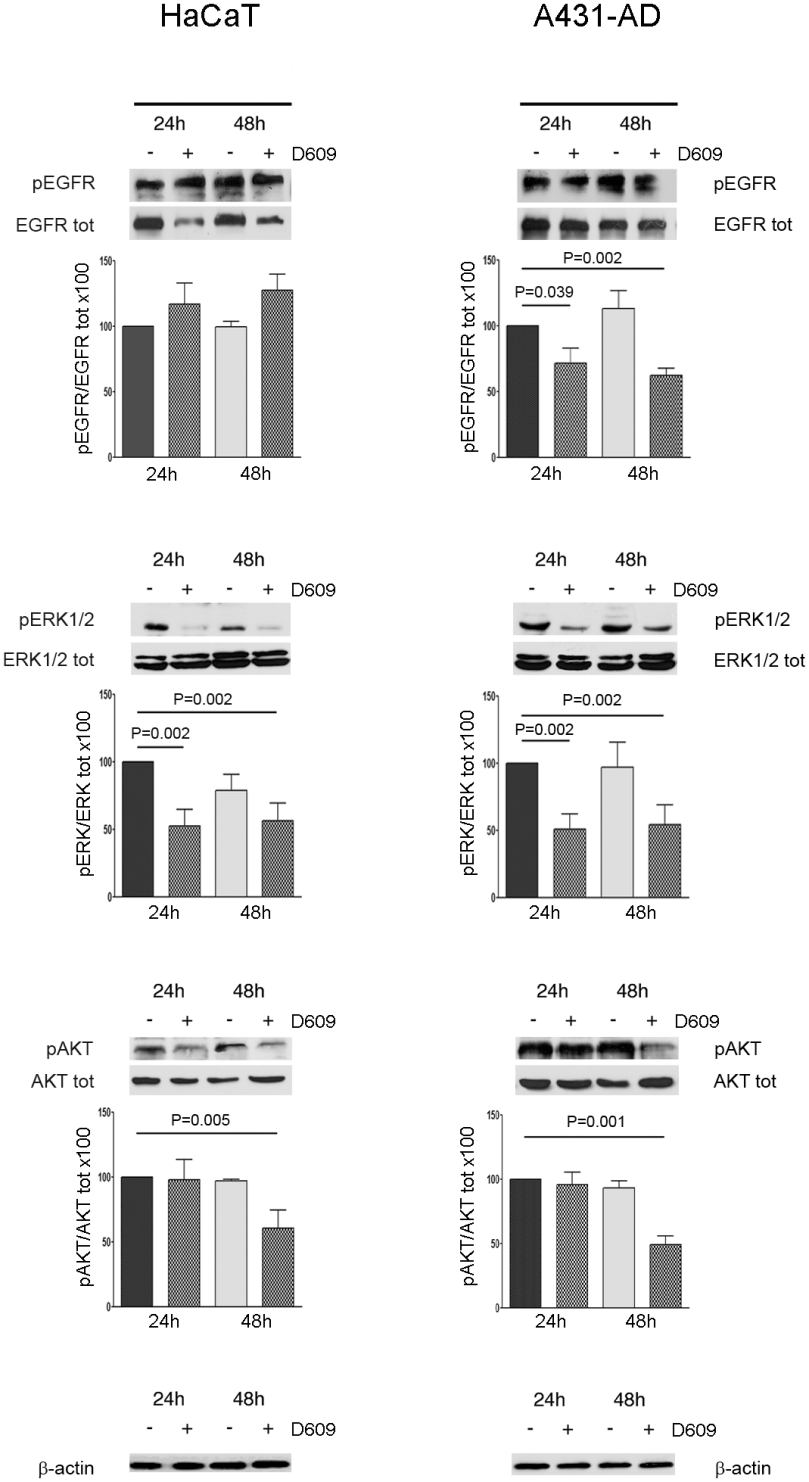
Effects of PC-PLC inhibition on EGFR, ERK and AKT phosphorylation in the HaCat and A431-AD cells. Representative Western blot analyses of total cell lysates from HaCaT (left panels) and A431-AD (right panels) cells cultured in the presence or absence of 50 μg/ml of D609. Cell lysates were immunoblotted with the following antibodies: pEGFR (Tyr1068), EGFR, pERK1/2 (Thr202/Tyr204), ERK 1/2, pAKT (Ser473), AKT and β-actin. β-actin was used as a quantitative loading control. Histograms below each panel represent the relative optical densities of phospho-protein levels normalized to the total protein level (mean values ± SD of three independent experiments) and are presented relative to the untreated sample at 24h. Statistical analyses were performed between treated samples (24h and 48 h) and untreated control sample at 24h, using the t-test.

As shown in Fig 3, quantitative Western blot analyses performed on total cell lysates showed that the level of EGFR phosphorylation (Y1068 residue) was substantially reduced by D609 in A431-AD cells at 24h (to about 72%, P = 0.039) and 48h (to about 62%, P = 0.002), while no effect was observed on the total level of EGFR protein. On the contrary, in HaCaT cells the phosphorylated Y1068 form of EGFR was not affected by D609, whereas the total level of the EGFR protein decreased (Fig 3, top pair of panels). In both cell lines the relative phosphorylation of ERK1/2 (pERK/total ERK ratio) was decreased to about 50% at 24h and persisted up to 48h of D609 exposure (Fig 3, middle pair of panels, P = 0.002). Compared with HaCaT, A431-AD cells showed a higher AKT content. A similar percent decrease in the relative AKT phosphorylation however occurred in the two cell lines at 48h (pAKT/total AKT ratio reduced to 50% in the HaCat, P = 0.005, and to 60% in the A431-AD cells, P = 0.001) but not at 24h of D609 treatment (Fig 3, bottom pair of panels).

Overall, these results supported the view that the PC-PLC activity could affect the EGFR signaling in the A431 cancer cells and have an impact on the mechanisms of MAPK- and AKT-mediated cell signaling cascades.

### Comparison of PC-PLC protein expression, activity and cellular localization in A431-AD and in A431-SPH

**Fig 4:**
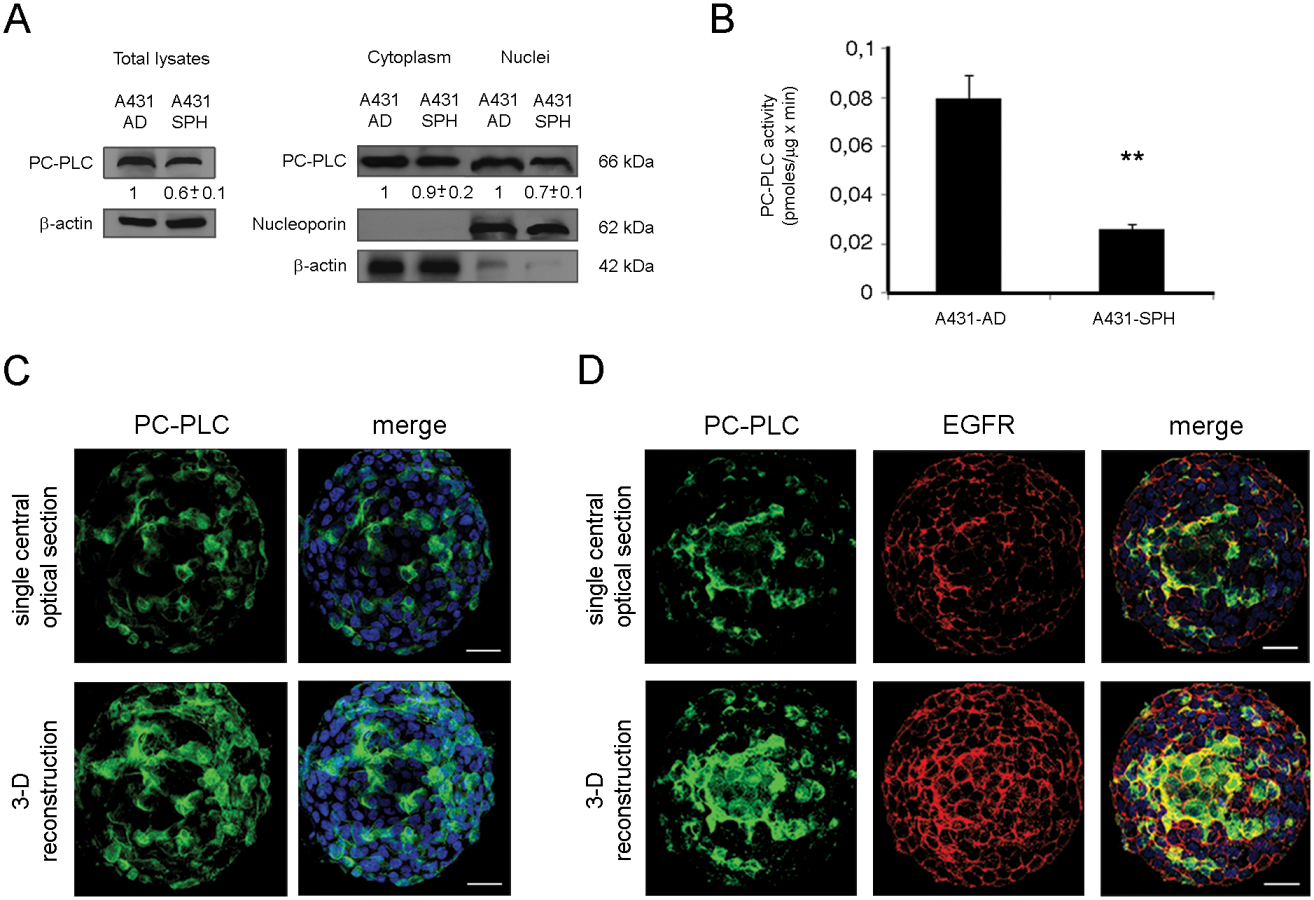
Comparison of PC-PLC protein expression, activity and cellular localization in A431-AD and in a model of cancer-initiating A431-SPH. **A)** Western blot analysis of PC-PLC relative levels in total cell lysates (left panels) and in the cytoplasmic and nuclear fractions (right panels). Nucleoporin and actin were used to ensure the quality of fractions separation and protein quantitative loading control, respectively. Densitometric analyses were performed and results (mean ± SD of three independent experiments) are shown as relative fold change of PC-PLC protein levels normalized to the actin (for total lysates and cytoplasmic fractions) or nucleoporin (for nuclear fractions) levels. Data are presented relative to the A431-AD cell line. **B)** PC-PLC activity (mean ± SD, n = 3) measured by Amplex Red assay in total cell lysates. P = 0.002. **C, D)** CSLM analyses of the single central optical section (top panels) and Z-projection of 25 optical sections taken from the bottom to the edge (3-D reconstruction, bottom panels) of the A431 sphere cells cultured for 72 hours, then fixed, permeabilized and stained for PC-PLC (green), EGFR (red) and nuclei (blue) detection. Scale bar, 40 μm.

The A431-SPH cells were first analyzed for PC-PLC protein expression level, subcellular localization and activity. Comparison of Western blot analyses of total cell lysates and subcellular fractions of A431-AD and A431-SPH cells showed that the overall PC-PLC content was lower in the spheres (fold change 0.6 ± 0.1), and its subcellular distribution indicates that the main differences between A431-AD and A431-SPH rely exclusively on the nuclear fraction (fold change in A431-SPH 0.7 ± 0.1) (Fig 4A). In substantial agreement with the difference in the PC-PLC protein level, Amplex Red assays showed a significant 2.8 ± 0.2 fold lower enzymatic activity in A431-SPH than in A431-AD cells (Fig 4B, P = 0.002). CLSM analyses on fixed and permeabilized A431-SPH cells showed that PC-PLC was expressed to a substantial level only in a subset of cells constituting the sphere (Fig 4C). Since it is well known that the A431-AD cells are characterized by over-expression of EGFR, we also evaluated the expression of this receptor on the A431-SPH cells. Interestingly, the PC-PLC^+^ subset also showed a remarkable degree of co-localization between the enzyme and the EGFR receptor (Fig 4D), suggesting that PC-PLC might play an important role in regulating the EGFR-driven oncogene signaling in this cellular component.

### Cytostatic and cytotoxic effects of PC-PLC inhibition on the A431-SPH cells proliferation and survival

**Fig 5:**
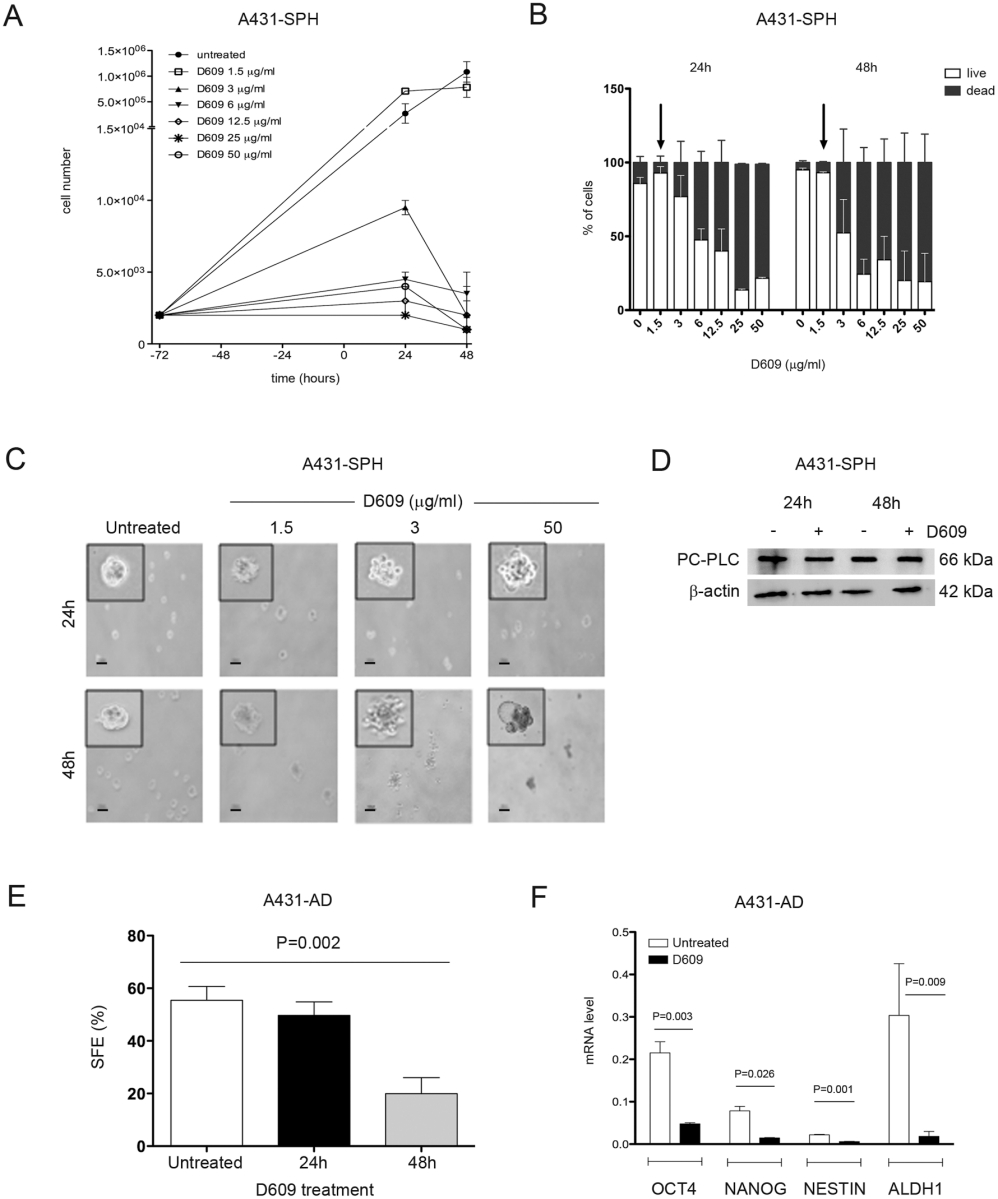
Cytostatic and cytotoxic effects of D609 on A431-SPH cells. Cells were seeded 72 hours before adding different doses of D609 at t = 0 (● = untreated cells; □ = 1.5 μg/ml, ▲ = 3 μg/ml, ▼ = 6 μg/ml, ◇ = 12.5 μg/ml, * = 25 μg/ml and ○ = 50 μg/ml) and monitored for 24h and 48 h afterwards. Cell counting (mean ± SD, n = 3) of total present cells (**A**) and of live (white columns) and dead (black columns) cells (**B**) measured by Trypan blue exclusion test and by automated cell counter, as described in the Material and Methods section. Arrows in the panel B indicate the dose of D609 used for the Western blot analyses reported in D. **C)** Evaluation of the effect of PC-PLC inhibition on A431-SPH cell morphology and death. Images were taken on untreated cells or after 24h and 48h of D609 exposure at the indicated doses. Scale bars, 100 μm. Inserts show a 100x magnification. **D)** Representative Western blot analyses (of 3 independent experiments) of PC-PLC protein expression in total lysates of A431-SPH cells, cultured in the presence or absence of 1.5 μg/ml D609 for the indicated times; β-actin was used as a quantitative loading control. **E**) Sphere Formation Efficiency (SFE) of A431-AD cells untreated (white column) or treated with 50 μg/ml D609 for 24h (black column) or 48h (grey column) (mean ± SD; n = 4 at T0 and 24h; n = 7 at 48h). Statistical analyses were performed using one-way ANOVA, P = 0.002. **F**) Real Time PCR analyses of stemness markers OCT4, NANOG, NESTIN and ALDH1 expressed by A431-AD cell lines either untreated (white columns) or in the presence of D609 (50 μg/ml, black columns) for 48h. Statistical analyses were performed using t-test (OCT4, P = 0.003; NANOG, P = 0.026; NESTIN, P = 0.001; ALDH1, P = 0.009).

To assess whether D609 has an active role on the TIC sub-population in squamous cancer, we evaluated the effects of D609 treatment on A431-SPH cells. Fig 5A shows that D609 was extremely potent in inhibiting the proliferation of A431-SPH cells; indeed at 3 μg/ml, i.e. at a dose 16-fold lower than that effective on parental A431-AD cells and HaCaT keratinocytes (for comparison see Fig 2B), D609 was already able to almost totally block the A431-SPH growth.

Differently from what we reported above for the A431-AD and for the keratinocytes cell lines, the inhibition of PC-PLC induced both cytostatic and cytotoxic effects in the A431-SPH cells. Starting from the 3 μg/ml D609 dose the A431-SPH appeared to undergo a cell death process, about 50% of dead cells being detected after 48h of treatment (Fig 5B). As shown in Fig 5C, morphological changes typical of cell death, such as blebbing and cell shrinkage, were observed in the spheres treated with 3 μg/ml D609. At a lower D609 dose that did not induce any substantial cytotoxic effect on A431-SPH cells (1.5 μg/ml, IC50 1.6 μg/ml), no alterations were detected in the PC-PLC expression level of A431-SPH cells (Fig 5D), as already observed in HaCaT and A431-AD cells treated with the 50 μg/ml dose of D609 (see Fig 2A). To investigate whether the cytotoxic effect was due to the growth in suspension, we tested the D609 effect on A431 spheroids, obtained by growing A431-AD cells in low attachment conditions and in presence of 10% FCS [40]. No cytotoxic effects were induced on A431 spheroids exposed to D609 for 48h at doses lower than 12.5 μg/ml (S3 Fig), a level 4-fold higher than that sufficient to induce a strong cytotoxicity on A431-SPH cells (3 μg/ml).

At a dose as low as 1.5 μg/ml, D609 failed to induce in A431-SPH cells any substantial change in the phosphorylation/activation status of ERK1/2 and AKT (S4 Fig). However, the EGFR expression and its phosphorylation level appeared to be increased at 48h, independently of D609 treatment (S4 Fig), suggesting that this receptor might play an important role in the A431 sphere self-renewal.

Overall, these results demonstrate that PC-PLC inhibition displays potent effects on the integrity and survival of A431 sphere cells, which have been shown to be characterized by the presence of a tumor initiating cells subset.

### PC-PLC regulates the A431 cell line stemness potential

To evaluate the effects of D609 on the A431-AD cell line ability to grow in suspension as SPH and to modulate the expression level of stem cell markers, A431 cells were exposed for different time intervals (24h and 48h) to this inhibitor (50 μg/ml), then seeded to form spheres for 7 days. A431-AD cells, which had been exposed to D609 for 24h, showed an only slight, if any, reduction in the sphere forming efficiency (SFE = 49.7 ± 5.1% compared with 55.5 ± 5.2% measured in untreated cells, Fig 5E). A much stronger effect was instead well evident in cells exposed for 48h to D609, in which SFE (19.9 ± 6.1%) was 2.8-fold lower than that of untreated cells and 2.5-fold lower than that observed 24h after treatment (P = 0.002) (Fig 5E). Real Time PCR performed on mRNA from A431-AD cells, treated with D609 for 48h, showed that the drug induced significant decreases in the expression levels of stem genes such as OCT4, NANOG, NESTIN and ALDH1 (P = 0.003, P = 0.026, P = 0.001 and P = 0.009, respectively) (Fig 5F). When the expression levels of these genes were evaluated in A431-SPH treated with a dose of D609 as low as 1.5 μg/ml, no significant changes were detected as compared to the untreated cells (data not shown).

### Role of PC-PLC in proliferation, survival and stemness potential of the CaSki cell line

**Fig 6:**
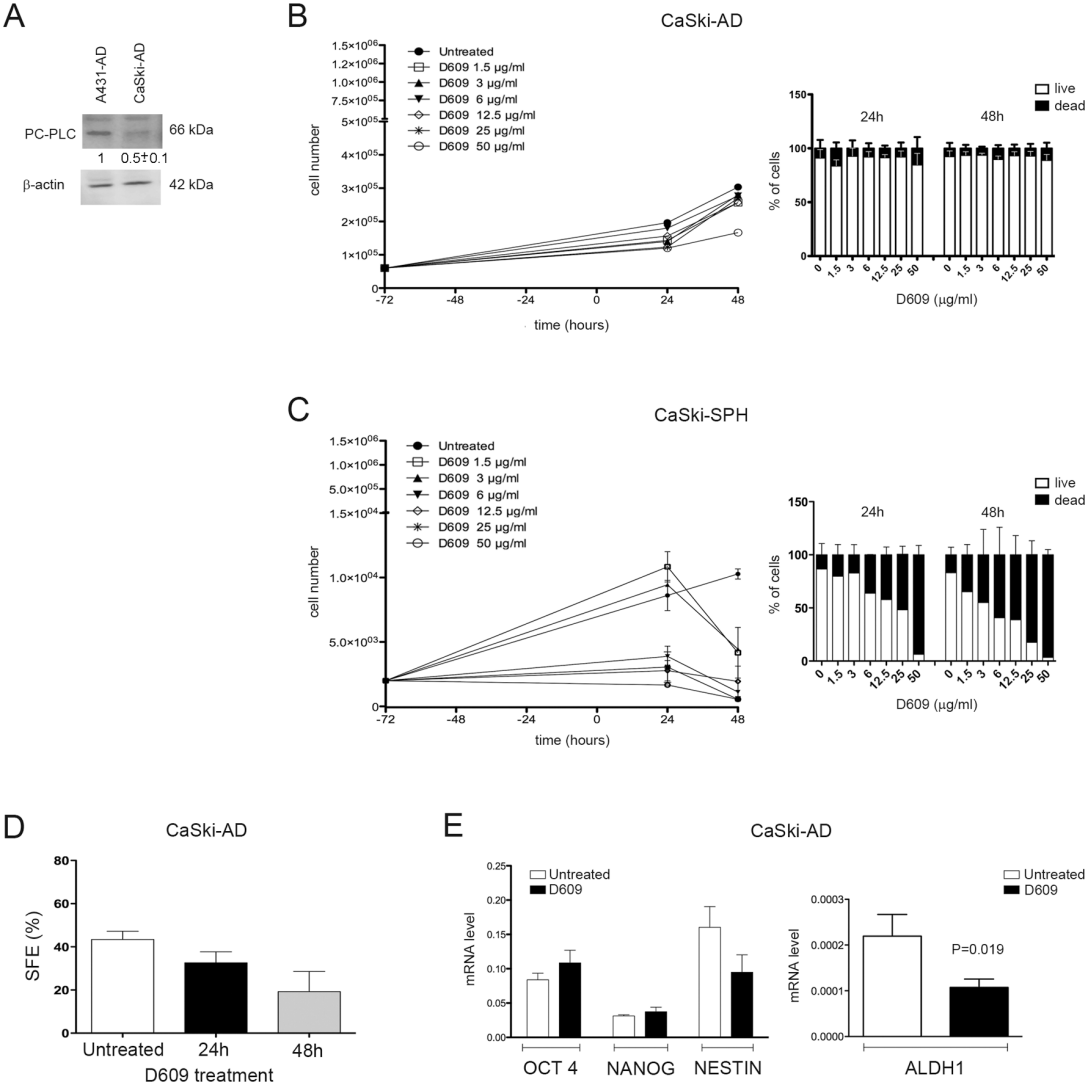
Comparison of PC-PLC protein expression in A431-AD and CasKi-AD cells and effects of D609 on cell proliferation and stemness potential of CaSki cells. **A)** Western blot analyses of the relative PC-PLC protein expression in A431-AD and CaSki-AD total cell lysates. β-actin was used as quantitative loading control. Densitometric analyses were performed and results are shown as relative fold change of PC-PLC protein levels normalized to the actin level. Data represent the mean (± SD) of three independent experiments, and are presented relative to the A431-AD cell line. **B**) Proliferation assays performed on CaSki-AD cells seeded 72 hours before adding different doses of D609 at t = 0 (● = untreated cells; □ = 1.5 μg/ml, ▲ = 3 μg/ml, ▼ = 6 μg/ml, ◇ = 12.5 μg/ml, * = 25 μg/ml and ○ = 50 μg/ml) and monitored for 24h and 48h afterwards. Cell counting (mean ± SD, n = 3) of total present cells (left panel) and of live (white columns) and dead (black columns) cells (right panel) measured by Trypan blue exclusion test and by automated cell counter, as described in the Material and Methods section. **C**) CaSki-SPH cells were seeded 72 hours before adding different doses of D609 at t = 0 (● = untreated cells; □ = 1.5 μg/ml, ▲ = 3 μg/ml, ▼ = 6 μg/ml, ◇ = 12.5 μg/ml, * = 25 μg/ml and ○ = 50 μg/ml) and monitored for 24h and 48h afterwards. Cell counts (mean ± SD, n = 3) of total present cells (left panel) and of alive (white columns) and dead (black columns) cells (right panel) measured by Trypan blue exclusion test. **D**) Sphere Formation Efficiency (SFE) of CaSki-AD cells untreated (white column) or treated with 50 μg/ml D609 for 24h (black column) or 48h (grey column) (mean ± SD; n = 2). Statistical analyses were performed using one-way ANOVA, P = 0.064. **E)** Real Time PCR analyses of the stemness markers OCT4, NANOG, NESTIN (left panel) and ALDH1 (right panel) expressed by the CaSki-AD cell line either untreated (white columns) or in the presence of D609 (50 μg/ml, black columns) for 48h. Statistical analyses were performed using t-test. (OCT4, P = 0.301; NANOG, P = 0.413; NESTIN, P = 0.173; ALDH1, P = 0.019).

PC-PLC protein expression, detected by Western Blot analyses, was much lower in CaSki-AD cells (fold change 0.5 ± 0.1) than in A431-AD cells (Fig 6A). Even though the proliferation rate of this cell line was lower than that of A431-AD cells, the inhibition of PC-PLC by D609 was able to induce a substantial cell growth arrest (55% of reduction) under appropriate conditions, i.e at the dose of 50 μg/ml and after 48h of treatment (Fig 6B, left panel). This anti-proliferative effect was similar to that observed in the HaCaT cell line exposed to D609. As indicated by the Trypan blue exclusion assay, the inhibition of PC-PLC did not exert any cytotoxic effect on CaSki-AD cells (Fig 6B, right panel). As previously shown by our group, CaSki cells were also able to grow in suspension thus forming spheres but, in comparison with the A431, the CaSki cell line tumor-initiating potential was much lower [5]. CaSki-SPH were exposed to D609 under the same conditions used for the previous experiments on A431-SPH. Fig 6C (left panel) shows that D609 at the dose of 3 μg/ml, the same dose effective in A431-SPH, was once again extremely potent in inhibiting the proliferation of CaSki-SPH cells (IC50 corresponding to 1.2 μg/ml), also inducing a strong cytotoxic effect (Fig 6C, right panel). As for A431-AD cells, we evaluated the stemness potential of CaSki-AD cells after exposure to D609 (50 μg/ml). After 24h of treatment CaSki-AD cells showed a small decrease in the sphere forming efficiency (SFE = 32.6 ± 7.2% compared with 43.4 ± 7.2% measured in untreated cells). A clear, although only border-line significant effect was detected in cells exposed to D609 for 48h, in which SFE (19.2 ± 13.3%) was 2.3-fold lower than that of untreated cells, and 1.7-fold lower than that observed after 24h of treatment (P = 0.064) (Fig 6D). Real Time PCR performed on untreated CaSki-AD mRNA revealed low expression levels of stem-related markers (Fig 6E). Real Time PCR carried out 48h after D609 treatment showed that the drug did not significantly change the expression level of OCT4 and NANOG stem genes, but it was associated with a significant reduction in ALDH1 expression (P = 0.019) and also with a trend towards a decrease in NESTIN.

## Discussion

Although some potentially interesting new drugs have been identified by high-throughput screening [41,42], no specific therapies have been found to definitely target the cancer stem cells population [13,43–45], hence any therapeutic approach selective for these cells represents a new challenge. In this paper we show that the PC-PLC competitive inhibitor D609, tested on A431 and on another SCC cell line, CaSki, displays a restricted and not previously reported activity on the subset of tumor initiating cells. In addition, D609 exerts, at very low doses, a strong toxic activity on the sphere-forming cell population, suggesting its potential therapeutic application as antitumor drug.

Although not yet cloned, neutral active PC-PLC isoforms responsible for phosphatidylcholine hydrolysis into phosphocholine and diacylglycerol have been recognized to be active and localized to plasma membrane in a variety of mammalian cells [22–34,46], and we and others highlighted in several studies the relevance of this enzyme in cell signaling through mitogen- and oncogene-activated protein kinase pathways [28,33,47,48]. In this study we first investigated PC-PLC protein expression level and its role in cell proliferation and survival by comparing A431-AD cancer cells with HaCaT keratinocytes, as models for SCC and its normal counterpart, respectively. We showed that tumor transformation increased PC-PLC expression and enzymatic activity and mostly modified the subcellular distribution of this enzyme, by inducing its appearance in the nucleus, in addition to its association with membrane and cytoplasmic compartments also detected in the non-tumoral HaCaT keratinocytes. These results confirm previous studies obtained in our laboratory that showed different patterns of subcellular PC-PLC distribution in different tumoral and non-tumoral cell systems [23–26].

We previously demonstrated that PC-PLC inhibition by D609 (50 μg/ml) results into a long-standing loss of cell proliferation in all tumoral and mitogen-stimulated non-tumoral cells investigated in our laboratory [23,25,26,28], exerting cytostatic (not cytotoxic) effects very close to those reported in the present study. Here we found that D609 impairs the cell proliferation ability in all the AD cell lines tested, an effect that was more evident in the A431 than in the CaSki and HaCat cell lines. In squamous carcinoma, the EGFR gene amplification, the cross-talk between other members of the HER family and the interaction with viral proteins have been implicated in oncogene-driven processes and remain an active subject of investigation [39]. It is well known that the A431 cells are characterized by over-expression of EGFR, while spontaneously immortalized keratinocytes HaCaT cells have low constitutive EGFR activity, and that the major sites of phosphorylation of EGFR are the Y1068 and Y1173 tyrosine residues [49]. Moreover, in many cancers, a defect in the PI3K/AKT/mTOR and MAPK/ERK pathways plays a crucial role in the development and progression of the disease [50]. In this context, our results suggest that PC-PLC inhibition affects the proliferation of SCC cells by modulating EGFR, AKT and ERK phosphorylation. Indeed, in the A431 tumor cell line, the inhibition of PC-PLC enzymatic activity is associated with a substantial reduction in the phosphorylation/activation levels of these molecules. D609 exposure of HaCaT cells did not affect the phosphorylation of the EGFR Y1068 residue, although the total level of the protein was significantly reduced. This result could be explained by the possible role of D609 as a stress-inducer able to elicit ligand-independent EGFR internalization [51], thus reducing the constitutive expression of the receptor and, likely, to stimulate its degradation, as already reported by Cichocki and colleagues in HaCaT cells [52] and by our group in breast carcinoma [25]. Indeed, it has been proved that internalization of stress-induced EGFR is a requirement for its activation that results in a specific phosphorylation of EGFR-Y1068 residue [53] thus explaining why in HaCaT cells the phosphorylation level of the EGFR Y1068 was not affected by D609. It also cannot be excluded that other EGFR residues, which were not evaluated in this study, might be affected and contributed to the overall EGFR protein reduction in HaCaT cells.

Our previous studies supported that an enrichment of the membrane PC-PLC component can typically be found in association with overexpression of some specific receptors [25,32], while exogenous or endogenous agents responsible for receptor stimulation may induce an increase in the nuclear PC-PLC fraction [28]. Different patterns of PC-PLC distribution among cellular compartments may therefore reflect the capability of this enzyme to participate in membrane-triggered signaling mechanisms, as well as, at the nuclear level, with transcriptional events acting on cell proliferation. Accordingly, A431-AD cells, which showed higher proliferative potential than HaCaT keratinocytes, also exhibit a high nuclear PC-PLC content, while both cell types share high levels of PC-PLC expression on the plasma membrane. Therefore, we could speculate that tumor transformation may induce an increase in the intra-nuclear lipid content and in the activity of enzymes involved in lipid metabolism [54].

In the A431 sphere population the PC-PLC sub-cellular distribution was similar to that found in A431-AD cells, although the total protein content and enzymatic activity were lower. However, CLSM analyses showed that PC-PLC distribution in A431-SPH was heterogeneous, suggesting that the PC-PLC^+^ sub-population might be, on the contrary, enriched in PC-PLC expression and activity. Interestingly, the PC-PLC^+^ sub-population, showed an extensive co-localization between the enzyme and EGFR. These observations seem worth of further investigations to assess the functional role of PC-PLC in regulating EGFR signaling and the differential structural/ functional role of these cell sub-populations in the formation and growth of spheres in suspension, thus favoring TICs self-renewal.

In contrast to the reported higher resistance to drug treatment of tumor spheres as compared to adherent cells [55–59], A431- and CaSki-SPH cells appeared to be very sensitive to D609 treatment. Indeed, a D609 dose of 3 μg/ml, i.e. about 16-fold lower than that effective on the respective AD cancer cells (50 μg/ml), was able to restrain the sphere proliferation. Besides the dramatic drop in cell proliferation, D609 treatment also exerted an evident cell death effect. Spheroids derived from A431-AD cells showed an intermediate sensitivity between AD and SPH cells to D609 treatment, supporting the idea that the highest sensitivity of sphere cells to D609 might be due to cell intrinsic properties rather than being merely due to cell growth in non-adherent conditions. In order to investigate which cell fraction could be the most affected by D609 treatment, we treated the A431- and CaSki-AD cells with the 50 μg/ml dose of the inhibitor and then evaluated their capability to form spheres. Since the SFE was strongly reduced in A431 (about 64%) and to a less extent also in CaSki cells (about 56%) we concluded that, while the effect of D609 as inhibitor of cell proliferation was extended to the whole cell population, the effect on SFE was probably mainly restricted to the cell fraction with stem-like characteristics. This hypothesis was confirmed by the quantitative evaluation of some stemness markers, such as OCT-4, Nanog, Nestin and ALDH1 [60], whose expression resulted differentially reduced in A431 and CaSki cells treated with D609. It has been reported that D609 (18.8–56.3 μM) blocks cell survival and induces apoptosis in embryo rat neural stem cells (NSC) [61]. The effect we observed on A431 and CaSki sphere cells was already evident at an even lower dose (11.3 μM, i.e. 3 μg/ml) and it appeared even more drastic than on NSC. On the contrary, on adult neural progenitors growing as spheres, the effect of D609 was detected at a higher dose (100 μM) and resulted in blocked cell proliferation, without cell mortality, with reduction of the number and size of growing spheres [62]. The different sensitivity and response to D609 treatment between embryo and adult neural stem cells, suggest that our sphere models are more similar to the embryo stem cells responding to low doses of D609 with a strong mortality. In conclusion, while a cytostatic effect mostly occurred after D609 treatment of adherent cells, in SPH cells the inhibition of PC-PLC induced, at much lower doses, both cytostatic and cytotoxic effects.

The different cellular responses observed after D609 treatment of AD and SPH populations of A431 and CaSki cells could be related to multiple actions of this agent on different metabolic pathways. In fact, besides inhibiting PC-PLC, D609 has been reported to also inhibit the activity of sphingomyelin synthase (SMS) [38], the enzyme responsible for phosphocholine transfer from phosphatidylcholine to sphingomyelin, and may even activate ceramide synthase, with the consequent possible production of increased, apoptosis-inducing levels of ceramides. Therefore we tested the possible inhibition of SMS activity by treating A431-AD cells with the highest D609 dose used in our experimental conditions (50 μg/ml) and we did not observed any reduction in the SMS activity, confirming previous studies reported in the literature [38,63], and showing that both inhibition of SMS, and activation of ceramide synthase, generally require much higher D609 doses than those found to be cytotoxic for A431-SPH cells.

Overall, our results suggest that PC-PLC acts as a master regulator of sphere cell proliferation and survival and its inhibition might lead to the targeting of TICs in squamous carcinoma. Previous studies have shown that D609 induced a partial regression of different types of human tumor transplants in athymic mice [27] and, more recently, we found that a significant tumor growth delay in human ovarian cancer xenografts treated with D609 was associated with a reduction of the total choline-containing metabolites MRS signal (Canese R. et al., Proceedings International Society for Magnetic Resonance in Medicine, 2013). Furthermore, near infrared probes have been designed, capable to detect the presence of D609-sensitive PC-PLC activity in *in vivo* human prostate xenografts [64].

Although this body of evidence suggests a role of PC-PLC in the *in vivo* tumor growth, further investigations are warranted to elucidate selective effects of D609 on TIC cells in experimental models of human cancers *in vivo*.

Meanwhile, we can conclude that PC-PLC inhibition may represent a novel approach to selectively target TICs and accordingly may be proposed—in combination with conventional therapies—to simultaneously eradicate in SCC the bulk population of highly proliferating chemo-sensitive cells and the sub-population of most aggressive and chemo-resistant TICs.

## Supporting Information

S1 Fig Characterization of genomic and metabolic profiles of normal keratinocytes and SCC cell lines. Unsupervised Hierarchical Clustering of 20 samples (HaCaT n = 4, patient skin derived keratinocytes n = 5, A431 n = 3, SiHa n = 4 and CaSki n = 4) analyzed for gene expression. The genes with low variance across the arrays were filtered out imposing that the variance of the log-ratios for each gene compared to the median of all the variances yielded p<0.01. After filtering, 4914 genes were included in the unsupervised hierarchical analysis using centered correlation metric and average linkage.
(PDF)

S2 Fig Effects of the PC-PLC inhibitor D609 on Sphingomyelin Synthase (SMS) activity. Relative SMS activity, measured by TLC assay, (mean ± SD, n = 2) in total cell lysates of HaCaT and A431-AD cell lines after exposure to D609 (50 μg/ml, grey columns) for 24h or 48h, compared with untreated cells (white columns). Statistical analyses, performed using unpaired t-test, showed that the SMS activity was not significantly altered by D609 in either HaCaT or A431-AD cells.
(PDF)

S3 Fig Evaluation of the effects of PC-PLC inhibition on A431 spheroid cell morphology and death. A431 adherent cells were plated in low attachment conditions and cultured in the presence of 10% FCS to form spheroids. The upper panels show representative images of an untreated spheroid (left); a spheroid exposed for 24 hours to 25 μg/ml D609 (central) and an example of “dead spheroid” under conditions of 24h cell exposure to 50 μg/ml D609 (right). Scale bar, 100 μm. The bottom panel shows the effects of D609 on vitality/mortality of A431 spheroids. Cells were seeded 72 hours before adding different doses of D609 and monitored for 24h and 48h afterwards. Cell counts (mean ± SD, n = 3) of live (white columns) and dead (black columns) cells were measured by Trypan blue exclusion test.
(PDF)

S4 Fig Effects of PC-PLC inhibition on EGFR, ERK and AKT phosphorylation in A431- SPH cells. Representative Western blot analyses of total cell lysates from A431-SPH cells cultured in the presence or absence of 1.5 μg/ml of D609 for 24 or 48h. Cell lysates were immunoblotted with the following antibodies: pEGFR (Tyr1068), EGFR, pERK1/2 (Thr202/Tyr204), ERK1/2, pAKT (Ser473), AKT and β-actin. The latter was used as a quantitative loading control.
(PDF)

S1 Materials and Methods Experimental protocols used to performed the experiments reported in the supplemental figures. (PDF)
