# Supplementary material for: Inhibition of Phosphatidylcholine-Specific Phospholipase C Interferes with Proliferation and Survival of Tumor Initiating Cells in Squamous Cell Carcinoma
Source: PLoS One. 2015 Sep 24;10(9):e0136120. doi: 10.1371/journal.pone.0136120 (PMC4581859; doi:10.1371/journal.pone.0136120)
Supplement: S1 Fig — Unsupervised Hierarchical Clustering of 20 samples (HaCaT n = 4, patient skin derived keratinocytes n = 5, A431 n = 3, SiHa n = 4 and CaSki n = 4) analyzed for gene expression. The genes with low variance across the arrays were filtered out imposing that the variance of the log-ratios for each gene compared to the median of all the variances yielded p<0.01. After filtering, 4914 genes were included in the unsupervised hierarchical analysis using centered correlation metric and average linkage. (PDF) [file pone.0136120.s001.pdf]

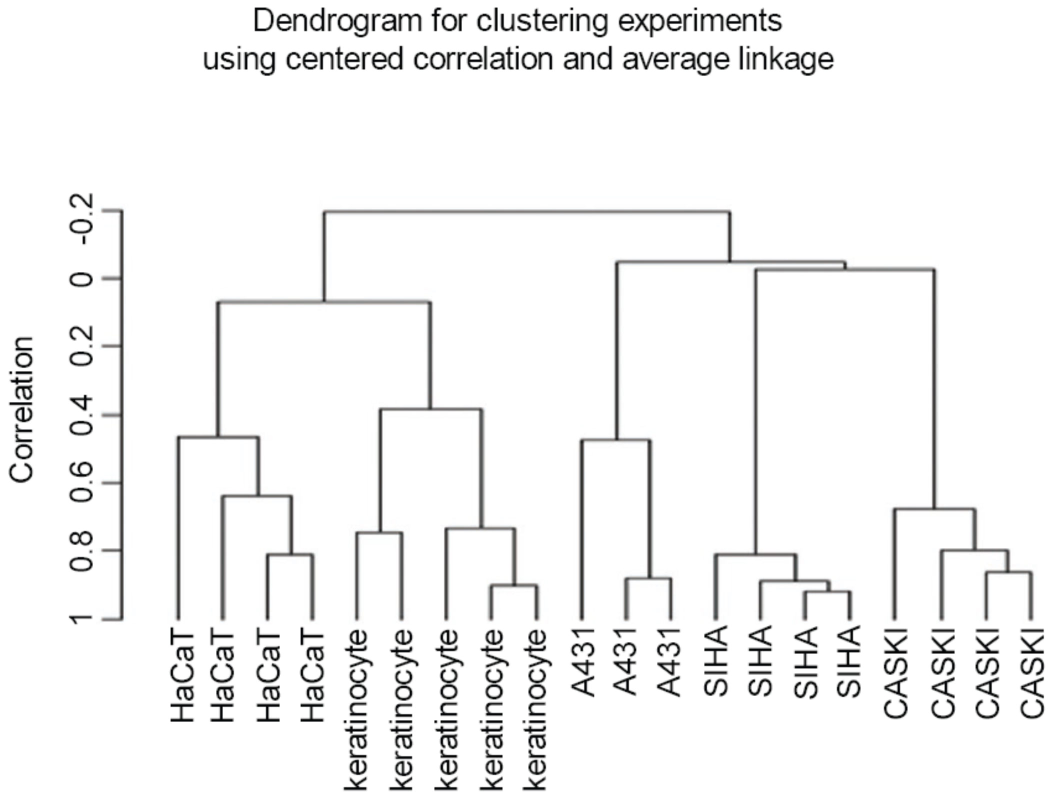

Unsupervised Hierarchical Clustering of 20 samples (HaCaT n=4, patient skin derived keratinocytes n=5, A431 n=3, SiHa n=4 and CaSki n=4) analyzed for gene expression. The genes with low variance across the arrays were filtered out imposing that the variance of the log-ratios for each gene compared to the median of all the variances yielded $p<0.01$. After filtering, 4914 genes were included in the unsupervised hierarchical analysis using centered correlation metric and average linkage.

Supplementary Figure 1
