# Supplementary material for: Inhibition of Phosphatidylcholine-Specific Phospholipase C Interferes with Proliferation and Survival of Tumor Initiating Cells in Squamous Cell Carcinoma
Source: PLoS One. 2015 Sep 24;10(9):e0136120. doi: 10.1371/journal.pone.0136120 (PMC4581859; doi:10.1371/journal.pone.0136120)
Supplement: S2 Fig — Relative SMS activity, measured by TLC assay, (mean ± SD, n = 2) in total cell lysates of HaCaT and A431-AD cell lines after exposure to D609 (50 μg/ml, grey columns) for 24h or 48h, compared with untreated cells (white columns). Statistical analyses, performed using unpaired t-test, showed that the SMS activity was not significantly altered by D609 in either HaCaT or A431-AD cells. (PDF) [file pone.0136120.s002.pdf]

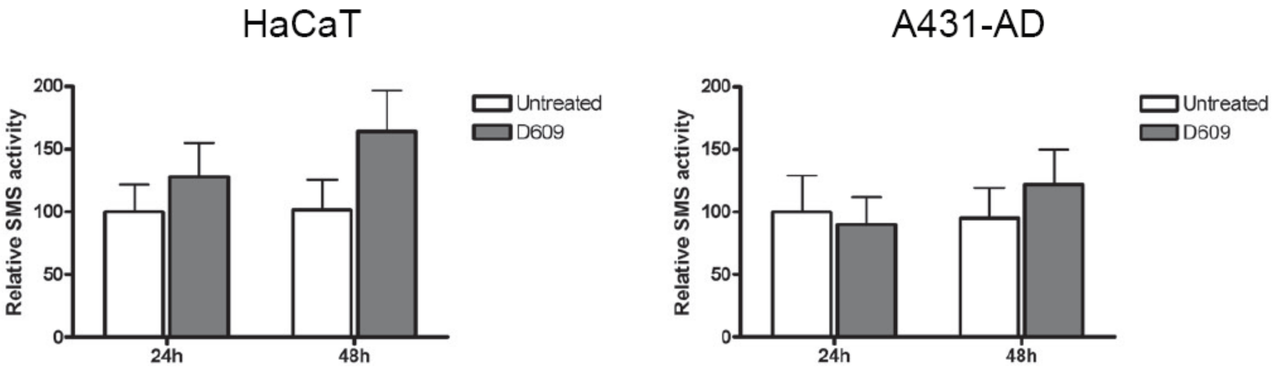

Effects of the PC-PLC inhibitor D609 on Sphingomyelin Synthase (SMS) activity.
Relative SMS activity, measured by TLC assay, (mean ± SD, n=2) in total cell lysates of HaCaT and A431-AD cell lines after exposure to D609 (50 µg/ml, grey columns) for 24h or 48h, compared with untreated cells (white columns). Statistical analyses, performed using unpaired t-test, showed that the SMS activity was not significantly altered by D609 in either HaCaT or A431-AD cells.

Supplementary Figure 2
