# Supplementary material for: Inhibition of Phosphatidylcholine-Specific Phospholipase C Interferes with Proliferation and Survival of Tumor Initiating Cells in Squamous Cell Carcinoma
Source: PLoS One. 2015 Sep 24;10(9):e0136120. doi: 10.1371/journal.pone.0136120 (PMC4581859; doi:10.1371/journal.pone.0136120)
Supplement: S3 Fig — A431 adherent cells were plated in low attachment conditions and cultured in the presence of 10% FCS to form spheroids. The upper panels show representative images of an untreated spheroid (left); a spheroid exposed for 24 hours to 25 μg/ml D609 (central) and an example of “dead spheroid” under conditions of 24h cell exposure to 50 μg/ml D609 (right). Scale bar, 100 μm. The bottom panel shows the effects of D609 on vitality/mortality of A431 spheroids. Cells were seeded 72 hours before adding different doses of D609 and monitored for 24h and 48h afterwards. Cell counts (mean ± SD, n = 3) of live (white columns) and dead (black columns) cells were measured by Trypan blue exclusion test. (PDF) [file pone.0136120.s003.pdf]

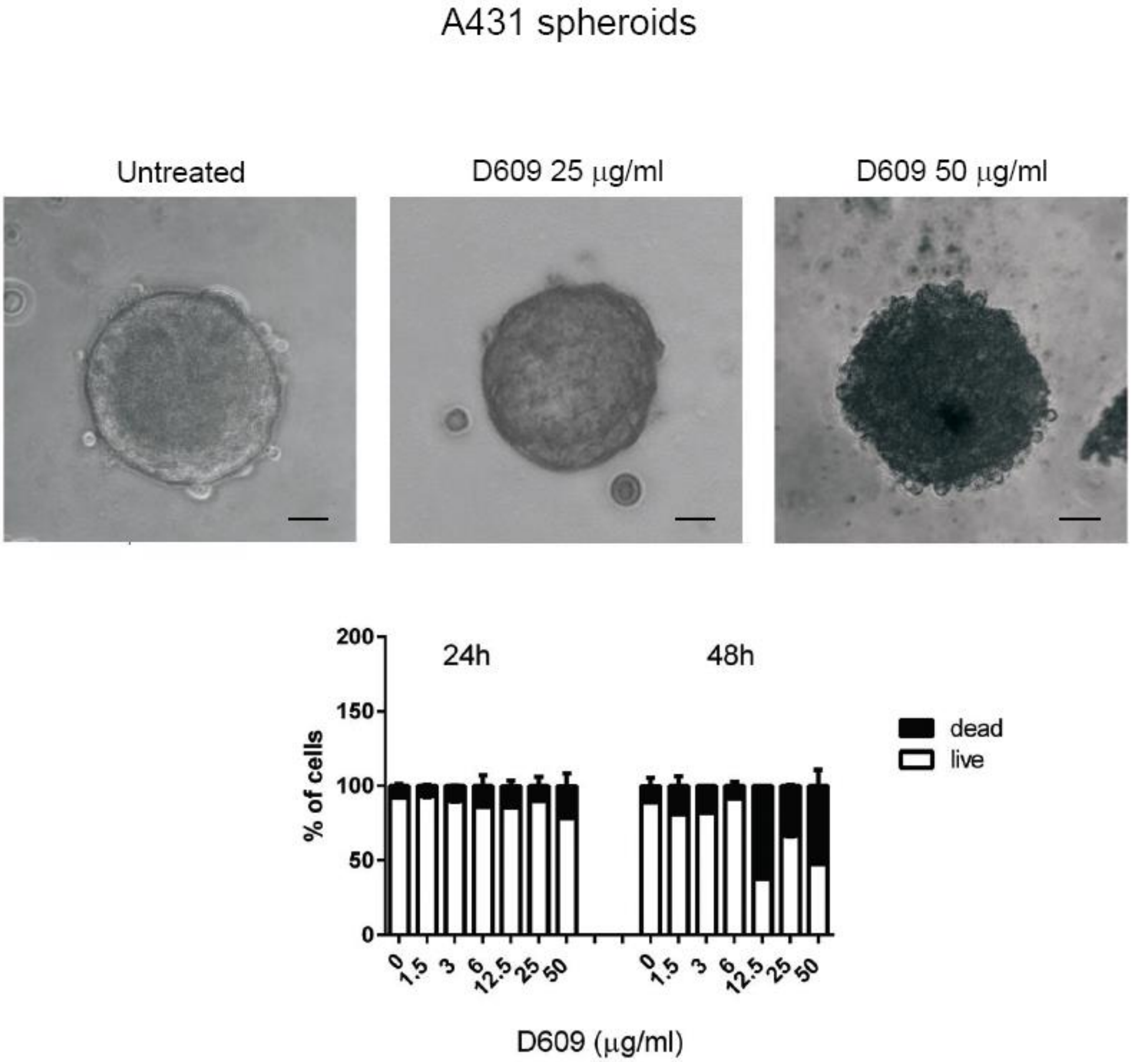

Evaluation of the effects of PC-PLC inhibition on A431 spheroid cell morphology and death. A431 adherent cells were plated in low attachment conditions and cultured in the presence of 10% FCS to form spheroids. The upper panels show representative images of an untreated spheroid (left); a spheroid exposed for 24 hours to 25 µg/ml D609 (central) and an example of “dead spheroid” under conditions of 24h cell exposure to 50 µg/ml D609 (right). Scale bar, 100 µm. **Effects of D609 on vitality/mortality of A431 spheroids.** Cells were seeded 72 hours before adding different doses of D609 and monitored for 24h and 48h afterwards. Cell counts (mean ± SD, n = 3) of live (white columns) and dead (black columns) cells were measured by Trypan blue exclusion test.

Supplementary Figure 3
