# Supplementary material for: Inhibition of Phosphatidylcholine-Specific Phospholipase C Interferes with Proliferation and Survival of Tumor Initiating Cells in Squamous Cell Carcinoma
Source: PLoS One. 2015 Sep 24;10(9):e0136120. doi: 10.1371/journal.pone.0136120 (PMC4581859; doi:10.1371/journal.pone.0136120)
Supplement: S4 Fig — Representative Western blot analyses of total cell lysates from A431-SPH cells cultured in the presence or absence of 1.5 μg/ml of D609 for 24 or 48h. Cell lysates were immunoblotted with the following antibodies: pEGFR (Tyr1068), EGFR, pERK1/2 (Thr202/Tyr204), ERK1/2, pAKT (Ser473), AKT and β-actin. The latter was used as a quantitative loading control. (PDF) [file pone.0136120.s004.pdf]

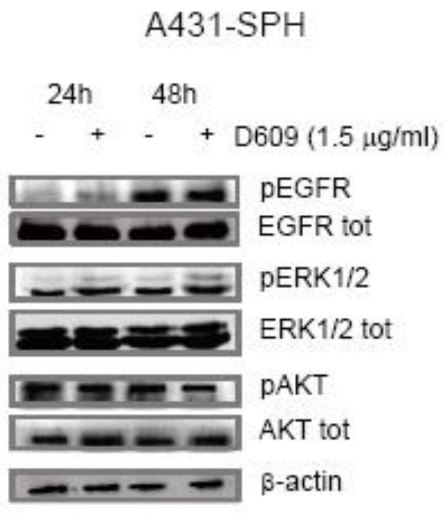

Effects of PC-PLC inhibition on EGFR, ERK and AKT phosphorylation in A431-SPH cells. Representative Western blot analyses of total cell lysates from A431-SPH cells cultured in the presence or absence of 1.5 μg/ml of D609 for 24 or 48h. Cell lysates were immunoblotted with the following antibodies: pEGFR (Tyr1068), EGFR, pERK1/2 (Thr202/Tyr204), ERK1/2, pAKT (Ser473), AKT and β-actin. The latter was used as a quantitative loading control.

Supplementary Figure 4
