# Supplementary material for: Inhibition of Phosphatidylcholine-Specific Phospholipase C Interferes with Proliferation and Survival of Tumor Initiating Cells in Squamous Cell Carcinoma
Source: PLoS One. 2015 Sep 24;10(9):e0136120. doi: 10.1371/journal.pone.0136120 (PMC4581859; doi:10.1371/journal.pone.0136120)
Supplement: S1 Materials and Methods — (PDF) [file pone.0136120.s005.pdf]

Supplementary Material and Methods

Unsupervised Hierarchical Clustering Experiments

Total RNA was isolated from 20 samples using Qiazol (Qiagen) reagent. After a clean-up treatment with RNAeasy kit following the manufacture's recommendations (Qiagen, Valencia, CA) and with RNase-free DNase to remove contaminating genomic DNA, RNA integrity and purity was assessed by Bioanalyzer (Agilent). RNA samples were processed for microarray hybridization by the Functional Genomics core facility at the Fondazione INT (Milan). Briefly, 300 ng of total RNA was reverse transcribed, labeled with biotin and amplified overnight (14 hours) using the Illumina RNA TotalPrep Amplification kit (Ambion) according to manufacturer's protocol. 1 µg of the biotinylated cRNA sample were mixed with the Hyb E1 hybridizatioin buffer containing 37.5% (w/w) formamide and then hybridized to Sentrix Bead Chip HumanRef8_v2 (Illumina, Inc., San Diego, CA) at 58 °C overnight (18 hours). The array represents over 24.000 bead types, each with a unique sequence derived from human genes in the National Centre for Biotechnology Information Reference Sequence and UniGene database. Array chips were washed with manufacturer's E1BC solution, stained with 1 µg/ml Cy3-streptavidine (Amersham Biosciences) and eventually scanned with Illumina BeadArray Reader. We collected primary data using the supplied scanner software and the following analyses were performed using the BeadStudio Version 3 software package.

Quantile normalization was used to correct experimental distortions. A detection threshold of $p<0.05$ was set for each gene and RNA. Probes detected in less than 50% of the samples were eliminated from the analysis. Analyses were performed using BRB-Array Tools v4.0 stable release developed by Dr. Richard Simon (NCI) and the BRB-Array Tools development team (EMMES Corp). The genes with low variance across the arrays were filtered out imposing that the variance of the log-ratios for each gene compared to the median of all the variances yielded $p<0.01$. After

filtering, 4914 genes were included in the unsupervised hierarchical analysis using centered correlation metric and average linkage.

Sphingomyelin Synthase (SMS) activity in A431-AD and in HaCaT cells

SMS activity was detected according to the method of Meng et al. (*Exp. Cell Res. 2004*). HaCaT and A431-AD cells were cultured in the presence or absence of D609 (50 µg/ml) for 24h and 48h and then homogenized in ice-cold lysis buffer (250 mM sucrose; 5 mM HEPES, pH 7.4; 1 mM PMSF and 20 µg/mL leupeptin) by 15 passages through a 27-gauge x0.5-inch needle. Unbroken cells and nuclei were removed by centrifugation at 1000 x g for 10 min at 4°C. Protein concentration was determined by the Bradford method using the Bio-Rad protein assay (Bio-Rad Laboratories) and tested for SMS activity as follows: 250 µg proteins were preincubated for 10 min at 37°C in a total final volume of 250 µL incubation buffer (50 mM Tris-HCl, pH 7.4, 25 mM KCl, 0.5 mM EDTA). The enzymatic reaction was started by the addition of 10 nmol Bodipy-TR (BTR)-ceramide and 60 nmol PC (Molecular Probes) and incubated for 30 min. The reaction was then stopped by the addition of 1 mL chloroform/methanol (1:1). Lipids were extracted and resolved by TLC in chloroform/methanol/acetic acid glacial/H2O (50:37.5:3.5:2). The formation of BTR-sphingomyelin was quantified by measuring the fluorescence intensity using a phosphoimager (Typhoon 9200, Amersham Biosciences).

Generation of A431 tumor spheroids

For spheroid generation A431 tumor cells were grown in RPMI 1640 (Gibco, Life Technologies) supplemented with 10% fetal calf serum (FCS) (Ivascu A et al., *J Biomol Screen*, 2006). 200 µl/well of cell suspensions at the density of 0.5×10^4 cells/ml were dispensed into 96-well round-bottomed plates (Corning, Life Sciences) using a multichannel pipette. To prevent cell attachment the plates were precoated with 50 µl 0.5% poly-HEMA (Sigma) in 95% ethanol and air dried at 37˚C for three

days. Plates were incubated at 37°C, in the presence of 5% CO_2, and after 24h of culture different doses of the PC-PLC inhibitor (D609) were added for 24h and 48h. Proliferation rates were monitored at 24h and 48h of treatment by counting live and dead cells by Trypan blue exclusion assay. The relative percentage of live and dead cells was calculated based on the sum of live and dead cells at each time point and dose.

Western blot analyses of A431 spheres

A431-SPH were seeded at $1x10^3$ cells/ml in Ultra Low Attachment T25 flask (Corning, Life Sciences) and after 24h of culture were treated with D609 (1.5 µg/ml) for 24 and 48h. Then, cells were lysed in the RIPA buffer (150 mM NaCl, 50 mM Tris-Cl, pH 7.5, 1% Nonidet P-40, 0.5% sodium deoxycholate, 0.1% SDS containing the complete protease inhibitor cocktail Hoffman-La Roche Ltd) with the adding of 2mM Na_3VO_4 and 1mM NaF. Protein concentrations were determined by Bradford's protein assay. Cell lysates (30 µg protein) were resolved by SDS-PAGE and blotted with different antibodies: pEGFR (Tyr1068), EGFR, pERK1/2 (Thr202/204), ERK1/2, pAKT (Ser473), AKT (all from Cell Signaling) and β-actin (Sigma).

Supplementary references:

Meng A, Luberto C, Meier P, Bai A, Yang X, Hannun YA, Zhou D. Sphingomyelin synthase as a potential target for D609-induced apoptosis in U937 human monocytic leukemia cells. Exp Cell Res. 2004; 292:385-92.

Ivascu A and Kubbies M: Rapid generation of single-tumor spheroids for high-throughput cell function and toxicity analysis. J Biomol Screen 11: 922-932, 2006.
